# Sideroflexin 3 is an α-synuclein-dependent mitochondrial protein that regulates synaptic morphology

**DOI:** 10.1242/jcs.194241

**Published:** 2017-01-15

**Authors:** Inês S. Amorim, Laura C. Graham, Roderick N. Carter, Nicholas M. Morton, Fella Hammachi, Tilo Kunath, Giuseppa Pennetta, Sarah M. Carpanini, Jean C. Manson, Douglas J. Lamont, Thomas M. Wishart, Thomas H. Gillingwater

**Affiliations:** 1 Centre for Integrative Physiology, University of Edinburgh, Hugh Robson Building, Edinburgh, EH8 9XD, UK; 2 Euan MacDonald Centre for Motor Neurone Disease Research, Chancellor's Building, University of Edinburgh, Edinburgh, EH16 4SB, UK; 3 Division of Neurobiology, The Roslin Institute and Royal (Dick) School of Veterinary Studies, University of Edinburgh, Edinburgh, EH25 9RG, UK; 4 Molecular Metabolism Group, University/BHF Centre for Cardiovascular Science, Queen's Medical Research Institute, University of Edinburgh, Edinburg, EH16 4TJ, UK; 5 MRC Centre for Regenerative Medicine, Institute for Stem Cell Research, The University of Edinburgh, Edinburgh, EH16 4UU, UK; 6 FingerPrints Proteomics Facility, College of Life Sciences, University of Dundee, Dundee, DD1 5EH, UK

**Keywords:** Alpha-synuclein, Sideroflexin 3, Neurodegeneration, Synapse, Mitochondria

## Abstract

α-Synuclein plays a central role in Parkinson's disease, where it contributes to the vulnerability of synapses to degeneration. However, the downstream mechanisms through which α-synuclein controls synaptic stability and degeneration are not fully understood. Here, comparative proteomics on synapses isolated from α-synuclein^−/−^ mouse brain identified mitochondrial proteins as primary targets of α-synuclein, revealing 37 mitochondrial proteins not previously linked to α-synuclein or neurodegeneration pathways. Of these, sideroflexin 3 (SFXN3) was found to be a mitochondrial protein localized to the inner mitochondrial membrane. Loss of SFXN3 did not disturb mitochondrial electron transport chain function in mouse synapses, suggesting that its function in mitochondria is likely to be independent of canonical bioenergetic pathways. In contrast, experimental manipulation of SFXN3 levels disrupted synaptic morphology at the *Drosophila* neuromuscular junction. These results provide novel insights into α-synuclein-dependent pathways, highlighting an important influence on mitochondrial proteins at the synapse, including SFXN3. We also identify SFXN3 as a new mitochondrial protein capable of regulating synaptic morphology *in vivo*.

## Abstract (precis)

**Summary:** Comparative proteomics revealed that mitochondrial proteins are a major target for α-synuclein. Sideroflexin 3 (SFXN3) was identified as one α-synuclein-dependent mitochondrial protein capable of altering synaptic morphology *in vivo*.

## INTRODUCTION

α-Synuclein is an abundant neuronal protein with a central role in the pathophysiology of Parkinson's disease. Abnormal accumulation of protein aggregates containing α-synuclein in Lewy bodies is a pathological hallmark of Parkinson's disease, and mutations and multiplications of *SNCA*, the gene encoding α-synuclein, have been linked with familial cases of the disease (Stefanis, 2012). The physiological and neurotoxic functions of α-synuclein have been associated with a variety of cellular processes, including neurotransmission, protein degradation and mitochondrial function (Lashuel et al., 2013; Stefanis, 2012). For example, α-synuclein has previously been shown to affect mitochondrial functions including complex I activity, oxidative stress and protein import pathways (Di Maio et al., 2016; Liu et al., 2009; Parihar et al., 2008).

At the level of the synapse, α-synuclein supports neurotransmission by promoting SNARE complex assembly and regulating synaptic vesicle recycling and mobility (Burré et al., 2014; Murphy et al., 2000; Scott and Roy, 2012). However, whereas increased levels of α-synuclein attenuate the neurodegenerative phenotype caused by deletion of CSP-α (also known as DNAJC5) (Chandra et al., 2005), both increased expression and deletion of α-synuclein impair synaptic functions (Burre et al., 2010; Cabin et al., 2002; Nemani et al., 2010). Therefore, the molecular mechanisms through which α-synuclein influences synaptic form and function remain unclear.

Here, we have undertaken a proteomics screen to identify molecular pathways and proteins acting downstream of α-synuclein in synapses, identifying synaptic mitochondria and the mitochondrial protein SFXN3 as an important target.

## RESULTS AND DISCUSSION

### Identification of novel α-synuclein targets at the synapse

**Fig. 1.**
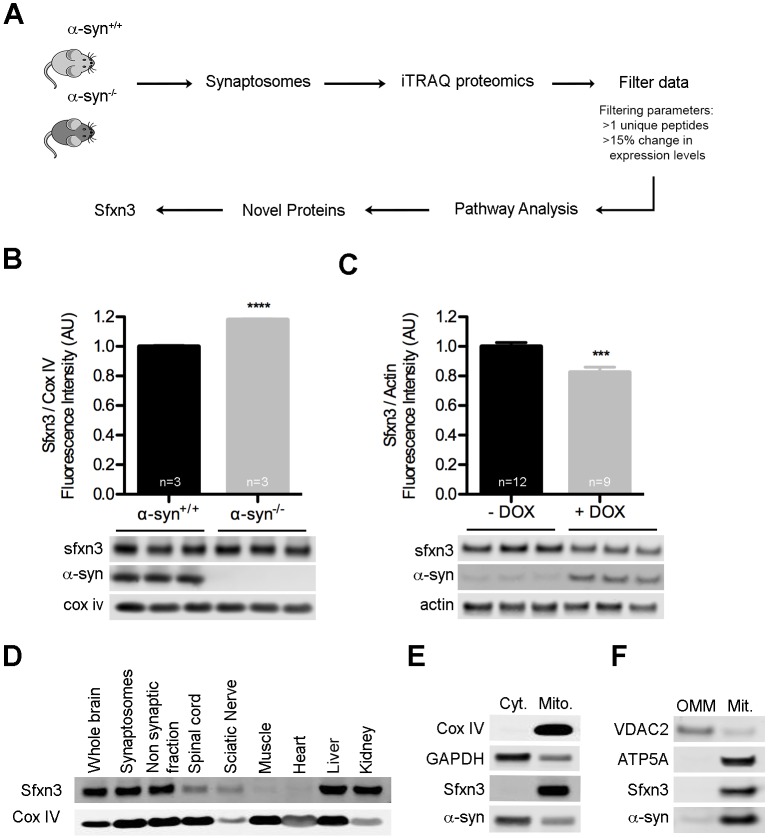
**Loss of α-synuclein at the synapse leads to widespread disruption of mitochondrial proteins, including SFXN3.** (A) Schematic overview of experimental design. iTRAQ, isobaric tag for relative and absolute quantitation. (B) SFXN3 protein levels were significantly upregulated in synaptosomes from α-syn^−/−^ compared to α-syn^+/+^ controls. ****P<0.0001 (unpaired *t*-test). Cox IV, loading control. (C) SFXN3 protein levels were significantly reduced in SH-SY5Y cells overexpressing WT α-synuclein induced by doxycycline (+DOX). ***P<0.001 (unpaired *t*-test). Actin, loading control. (D) Representative western blot showing SFXN3 expression across several tissues from an adult wild-type mouse. Cox IV was used as a mitochondrial marker. (E) SFXN3 was exclusively localized to mitochondrial (Mito), but not cytosolic (Cyt), fractions isolated from mouse brain. Cox IV was used as a mitochondrial marker and GAPDH as a cytosolic marker. (F) SFXN3 was exclusively localized to the inner mitochondrial membrane. Outer mitochondrial membrane (OMM) and mitoplasts (Mit.) were isolated from undifferentiated SH-SY5Y cells. VDAC2 was used as a marker for the OMM and ATP5A as a marker for the mitoplast fraction. All data are mean±s.e.m.

To uncover molecular mechanisms downstream of α-synuclein relevant for synaptic form and function, we quantified changes in the synaptic proteome of mice lacking α-synuclein (Fig. 1A). iTRAQ proteomics on synaptosomes from α-syn^+/+^ and α-syn^−/−^ mice identified 2615 individual proteins. Raw mass spectrometry data was filtered to leave only those proteins identified by at least two unique peptides and with expression levels consistently altered by ≥15% across two independent technical replicates (Table S1). The remaining 200 proteins were submitted to bioinformatics pathway analysis through ingenuity pathway analysis (IPA), DAVID and VarElect, revealing a striking enrichment of proteins (74 out of 200) belonging to mitochondrial pathways (Table 1; Table S1). This provides substantial experimental support for the hypothesis that α-synuclein has important physical and/or functional interactions with mitochondria (Haelterman et al., 2014; Nakamura, 2013). Further bioinformatics analysis identified 37 mitochondrial proteins not previously associated with α-synuclein or neurodegeneration (Fig. S1).

**Table 1.**
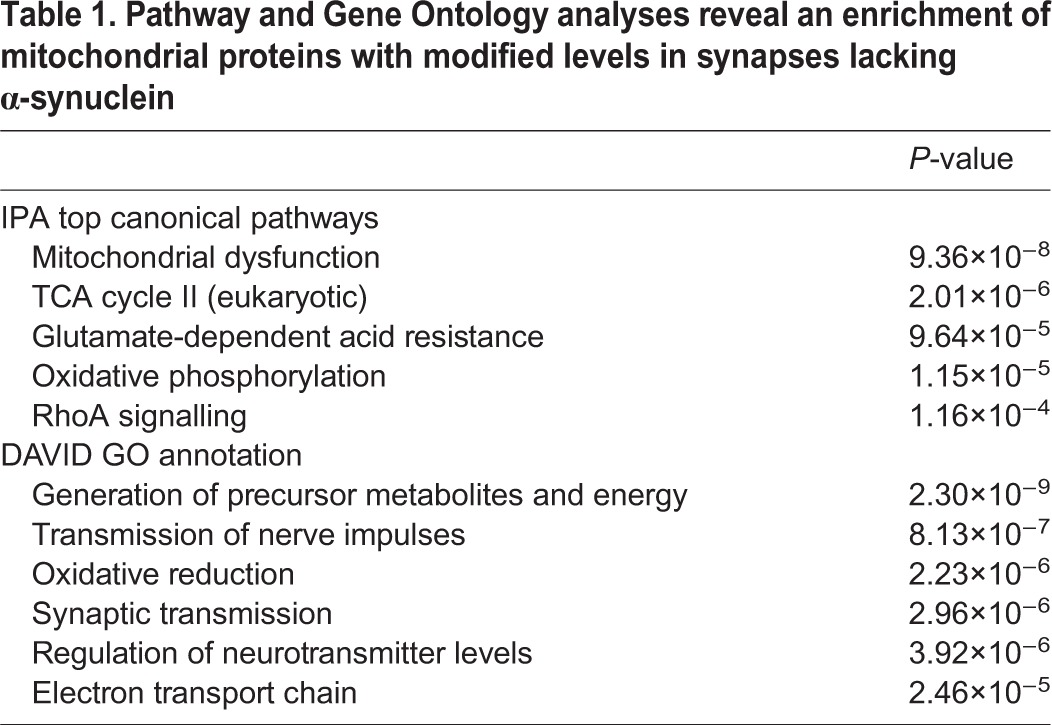
**Pathway and Gene Ontology analyses reveal an enrichment of mitochondrial proteins with modified levels in synapses lacking α-synuclein**

To select individual proteins from the shortlist of 37 to be prioritized for further analyses, we performed extensive literature searches to identify those with potential links to Parkinson's disease and/or neurodegeneration. Using this approach, sideroflexin 3 (SFXN3) was identified as a protein of particular interest. Studies have reported that levels of SFXN3 transcripts or protein were deregulated in the substantia nigra of Parkinson's disease patients and in rodents subjected to a 6-hydroxydopamine lesion (Charbonnier-Beaupel et al., 2015; Fuller et al., 2014; Simunovic et al., 2009). SFXN1, a protein from the same family, interacts with connexin 32 (also known as GJB1), mutations in which cause Charcot–Marie–Tooth disease (Bergoffen et al., 1993; Fowler et al., 2013), and reduced levels of SFXN1 protein have been reported in Alzheimer's disease (Minjarez et al., 2016). In addition, SFXN2 is upregulated in a dopaminergic cell line in response to rotenone treatment (Jin et al., 2007). SFXN3 belongs to a family of proteins (sideroflexins 1–5) that are putative iron transporters, with predicted transmembrane domains and mitochondrial localization (Fleming et al., 2001; Li et al., 2010). The functional role of these proteins, however, remains poorly understood.

Levels of SFXN3 protein were increased in synapses from α-syn^−/−^ mice (Fig. 1B), suggesting that SFXN3 expression is inversely correlated with α-synuclein. To confirm this, we overexpressed wild-type α-synuclein in stably transfected SH-SY5Y cells. Exposure of SH-SY5Y cells to doxycycline (DOX) for 24 h led to a significant increase in α-synuclein (5.78±0.81; *n*≥9; mean±s.e.m.; *P*=1.7×10^−6^ in *t*-test), accompanied by a significant decrease in levels of SFXN3 protein (Fig. 1C). Thus, SFXN3 levels are bi-directionally regulated by α-synuclein.

It is not clear how α-synuclein regulates levels of SFXN3. One possibility is that α-synuclein interferes with the import of SFXN3 into mitochondria, given that α-synuclein has been shown to inhibit the import of nuclear-encoded mitochondrial proteins through an interaction with TOMM20 (Di Maio et al., 2016). In support of this, we found several mitochondrial import proteins, such as TIMM10B, PAM16 and TOMM40, to be upregulated in α-syn^−/−^ mice compared to controls (Table S1).

### SFXN3 is a mitochondrial protein enriched in the inner mitochondrial membrane

SFXN3 is predicted to be a mitochondrial protein (Pagliarini et al., 2008), but experimental evidence confirming its tissue expression and subcellular localization is lacking. We used western blotting to analyse expression levels of SFXN3 protein in mice (Fig. 1D). SFXN3 was highly enriched in brain, both in synaptic and non-synaptic fractions, spinal cord and peripheral nerve. It was also present in liver and kidney, but was not detectable in skeletal or cardiac muscle.

Isolation of mitochondrial and cytosolic fractions from mouse brain confirmed that SFXN3 was expressed exclusively in mitochondria (Fig. 1E). Furthermore, differential extraction of mitochondrial outer membrane and mitoplasts of mitochondria from SH-SY5Y cells revealed that SFXN3 was localized to the mitoplast fraction (Fig. 1F). Given the presence of transmembrane domains in the SFXN3 protein (Li et al., 2010), we conclude that SFXN3 is preferentially localized to the inner mitochondrial membrane.

### Loss of SFXN3 does not influence mitochondrial bioenergetics

**Fig. 2.**
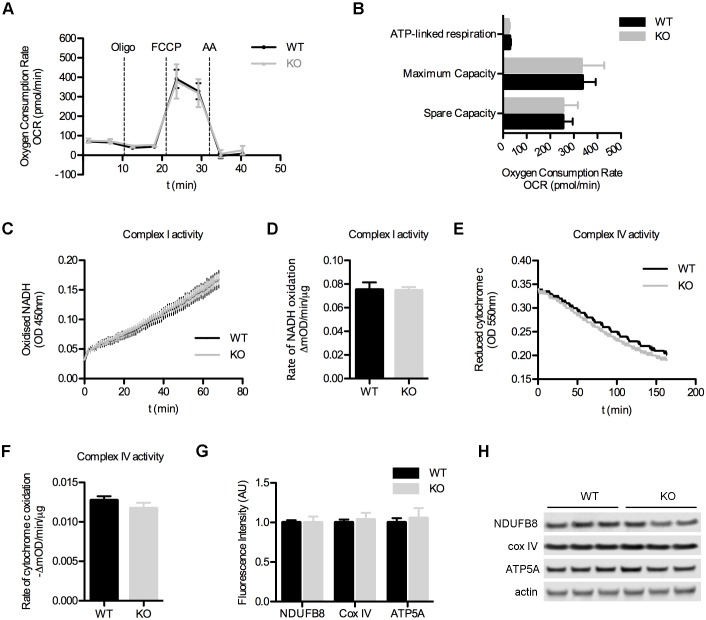
**Loss of SFXN3 does not affect mitochondrial bioenergetics.** (A) Identical oxygen consumption rates (OCR) in synaptosomes from WT and SFXN3-KO mice. Dashed vertical lines indicate the time of injection of Oligomycin (Oligo), FCCP (FCCP) and antimycin A (AA). *n*=3. (B) Bioenergetic parameters derived from results in A (see Materials and Methods). *n*=3, *P*>0.05 (unpaired *t*-test). (C) Complex I activity measured by tracking absorbance of oxidized NADH. *n*=3. (D) Rate of Complex I activity derived from results in C shows that the enzymatic activity of Complex I is not compromised in SFXN3-KO mice. *n*=3, *P*>0.05 (unpaired *t*-test). (E) Complex IV activity measured by tracking absorbance of reduced cytochrome *c*. *n*=3. (F) Rate of Complex IV activity derived from results in E shows that the oxidation of cytochrome *c* is not affected in SFXN3-KO mice. *n*=3, *P*>0.05 (unpaired *t*-test). (G,H) Western blotting showing unaltered levels of key ETC proteins in synaptosomes from SFXN3-KO mice. *n*=3, *P*>0.05 (unpaired *t*-test). All data are mean±s.e.m.

The localization of SFXN3 to the inner mitochondrial membrane prompted us to ask whether SFXN3 plays a role in canonical bioenergetic pathways, including oxidative phosphorylation. We isolated purified synaptosomes from wild-type (WT) and SFXN3-knockout (KO) mice and performed mitochondrial respiration assays using a Seahorse XF^e^24 Analyzer. Oxygen consumption rates (OCR) during basal respiration were similar between WT and SFXN3-KO mice (Fig. 2A). Accordingly, the fraction of ATP-linked respiration was comparable in WT and SFXN3-KO mice (Fig. 2B). Uncoupling of mitochondria, by using FCCP, induced equivalent spare and maximum respiratory OCR rates in WT and SFXN3-KO synaptosomes, indicating that synaptic mitochondria from SFXN3-KO mice are comparable to those from controls with respect to their ability to cope with short periods of high energetic demand (Fig. 2A,B). Thus, SFXN3 is not absolutely required for mitochondrial bioenergetics pathways, as mitochondrial respiration was unaffected by the absence of SFXN3.

To confirm that subtle effects on mitochondrial respiration prompted by reduced levels of SFXN3 were not being masked by compensatory mechanisms in enzymatic activity or abundance of other key electron transport chain (ETC) proteins, we performed enzymatic assays on immunocaptured Complex I and Complex IV (Fig. 2C,E). The rates of NADH and cytochrome *c* oxidation revealed identical enzymatic activities of Complex I and Complex IV, respectively, in WT and SFXN3-KO mice (Fig. 2D,F). Quantitative western blotting for ATP5A (a component of ATP synthase), and the Complex I and IV proteins NDUFB8 and Cox IV confirmed that no compensatory changes were occurring in these ETC proteins in SFXN3-KO mice (Fig. 2G,H).

### SFXN3 influences synaptic morphology at the *Drosophila* neuromuscular junction

**Fig. 3.**
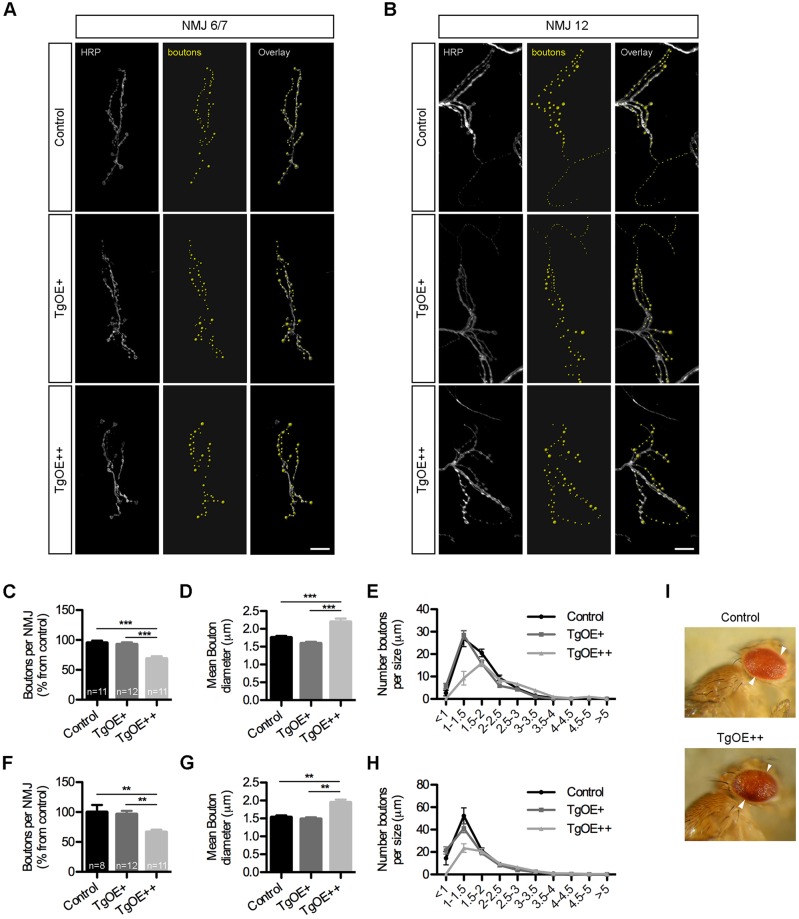
**Sfxn3 regulates synaptic morphology at the neuromuscular junction in *Drosophila*.** (A,B) NMJs on muscles 6 and 7 (A) and 12 (B) of control and transgenic larvae, with low (TgOE+) and high (TgOE++) overexpression of SFXN3. Note the reduction in boutons in TgOE++ NMJs (grey, anti-HRP; yellow, pseudo-coloured synaptic boutons). Scale bars: 10 μm. (C–H) Reduction in the number of boutons and increase in mean bouton diameter on NMJs from muscles 6 and 7 (C,D) and 12 (F,G) overexpressing SFXN3 (TgOE++). Results are mean±s.e.m. **P<0.01, ***P<0.001 (one-way ANOVA with Tukey post-test). Distribution of bouton diameter from NMJs on muscles 6 and 7 (E) and 12 (H) showing a specific reduction of small size (<1.5 μm) boutons in TgOE++ larvae. (I) Representative images of eyes from control and TgOE++ flies demonstrating no overt phenotype.

Given that SFXN3 was identified as a α-synuclein target in synaptic mitochondria, we wanted to establish whether SFXN3 contributes to pathways regulating synaptic stability. We obtained and mapped a UAS-driven P{EPgy2} *Drosophila* strain demonstrating insertion of the promoter in the correct direction and in the 5′UTR of our gene of interest, suggesting significant potential for overexpression of SFXN3 (*Drosophila* homologue of the sideroflexin gene; FlyBase annotation CG6812). The *Drosophila* UAS-Gal4 system was used to generate tissue-specific overexpression of SFXN3 in third-instar larval neurons using the pan-neuronal driver *elav-Gal4.* High-dose overexpression of SFXN3 (TgOE++) led to a significant reduction in the number of synaptic boutons, accompanied by an overall increase in mean bouton diameter, at the neuromuscular junctions (NMJs) from muscles 6 and 7 and from muscle 12 (Fig. 3). The most striking phenotype was a reduction in type II boutons innervating muscle 12. To confirm that these changes were occurring as a direct result of changes in SFXN3 levels, we repeated our analyses with a low dose overexpression of SFXN3 (TgOE+). These experiments confirmed no alterations in any of the neuromuscular parameters analysed (Fig. 3). Furthermore, analysis of eye morphology in TgOE++ flies showed no overt phenotype (Fig. 3I). The *Drosophila* eye is a robust and sensitive read-out for identifying neurodegeneration (Sanhueza et al., 2015), suggesting that overexpression of SFXN3 selectively influences synaptic morphology without initiating neurodegenerative cascades.

Taken together, our results demonstrate an important role for α-synuclein in regulating mitochondrial proteins at the synapse. We identify SFXN3 as one mitochondrial protein whose levels are bi-directionally regulated by α-synuclein and that is also capable of directly influencing synapses *in vivo*. Further studies will now be required to determine the extent to which SFXN3 acts as a key intermediary of α-synuclein-dependent synaptic pathology occurring in Parkinson's disease. Considering that α-synuclein itself is difficult to target from a therapeutic perspective (as a result of being intrinsically disordered, with diverse and poorly understood oligomeric states), targeting SFXN3 might ultimately represent an alternative strategy for maintaining synapses in Parkinson's disease.

## MATERIALS AND METHODS

### Reagents

All chemicals were purchased from Sigma Aldrich, except tissue culture reagents, the BCA assay, RIPA buffer and Halt protease inhibitor cocktail, which were from Thermo Scientific, and Percoll, which was from GE Healthcare Biosciences. Buffer compositions are in Table S2.

### Mice

C57Bl/6J (α-syn^+/+^) and C57Bl/6JOlaHsd (α-syn^−/−^) mice, carrying a natural α-synuclein deletion (Specht and Schoepfer, 2001), were obtained from University of Edinburgh breeding stocks. Sfxn3^tm1b(KOMP)Wtsi^ mice (SFXN3-KO mice; http://www.mousephenotype.org/data/genes/MGI:2137679) were obtained from the Wellcome Trust Sanger Institute Mouse Genetics Project as part of the nPad MRC Mouse Consortium, maintained on a C57Bl/6N background. Mice were of mixed gender and 2–4 months old. All work was covered by appropriate UK Home Office licenses.

### Drosophila

*Drosophila melanogaster* were raised on standard cornmeal food at room temperature. *elav-Gal4* and *GMR-Gal4* driver strains were used with stocks obtained from the Bloomington *Drosophila* stock center (IDs: y^1^ w^67c23^; P{EPgy2}CG6812^EY02703^; Canton-S). Crosses were maintained at 22°C for 24 h before removal of adults and embryos were incubated in a water bath to increase levels and activity of Gal4 proteins. Incubation temperatures were 25°C or 30°C for low dose (TgOE+) or high dose (TgOE++) overexpression experiments, respectively. For immunohistochemistry methods see Sanhueza et al. (2015). Images were analysed using IMARIS software to determine the number and transverse diameter of synaptic boutons. The investigator was blinded to the group allocation throughout.

### Isolation of synaptosomes and iTRAQ proteomics analysis

Isolation of crude synaptosomes from α-syn^+/+^ and α-syn^−/−^ mice was performed as described previously (McGorum et al., 2015; Wishart et al., 2012). Samples were homogenized in iTRAQ buffer and supernatant extracts from four mice per genotype were pooled. 100 μg protein was labelled with tags (114 and 116 α-syn^+/+^; 115 and 117 α-syn^−/−^) before injection into an Ultimate RSLC nano UHPLC system coupled to a LTQ Orbitrap Velos Pro (Thermo Scientific). Results were filtered to include proteins identified by at least two unique peptides with a ≥15% difference in levels across both α-syn^+/+^ vs α-syn^−/−^ comparisons.

Data were examined using bioinformatics tools: ingenuity pathway analysis (IPA; Ingenuity Systems, USA); Database for Annotation, Visualization and Integrated Discovery (DAVID; NIH, USA); and VarElect (LifeMap Sciences, USA). IPA was used to determine affected molecular pathways (Top Canonical Pathways). DAVID Functional Annotation was used to identify enrichment for gene ontology (GO) terms. VarElect was used to identify the fraction of data that included proteins associated with the terms ‘mitochondria’ and the group of terms ‘Parkinson's’, ‘Parkinson’, ‘PD’, ‘synuclein’ and ‘SNCA’.

### Tissue culture

SH-SY5Y cells were electroporated with a Tet-One™ plasmid (Clontech) encoding full-length human α-synuclein and a DOX-responsive clonal line was selected and maintained in Dulbecco's modified Eagle's medium (DMEM) supplemented with 10% fetal bovine serum (FBS), 1% penicillin-streptomycin, 1 mM sodium pyruvate and 2 µg/ml puromycin. For induction of α-synuclein expression, 24 h after plating cells were differentiated for 5 days in the presence of 10 µM retinoic acid and incubated in DMEM supplemented with 10 µg/ml of doxycycline for 24 h.

### Isolation and fractionation of mitochondria

Mitochondria were isolated from mouse brain using the Mitochondria Isolation Kit for Tissue (Abcam). Fractionation of mitochondria from undifferentiated SH-SY5Y cells was performed as described previously (Nishimura et al., 2014).

### Western blotting

Western blotting was performed as described previously (Eaton et al., 2013), using antibodies in Table S3.

### Mitochondrial respiration assays

Mitochondrial respiration assays were performed on purified synaptosomes, isolated from mouse forebrain using discontinuous Percoll gradients prepared in isolation medium, as previously described (Choi et al., 2009).

Synaptosomes (10 μg protein/well, ≥5 technical replicas per sample) were loaded into wells of XF^e^24 V7 (Seahorse Biosciences) uncoated plates. The plate was centrifuged at 2000 ***g*** for 20 min at 4°C, and 500 μl of incubation medium was added to each well prior to entry into the XF^e^24 Seahorse Analyzer (Seahorse Biosciences). Oxygen consumption rates (OCR) were measured in groups of two cycles of 1 min wait, 1 min mix, 3 min measurements, with an injection between each 2 cycles. Each well was sequentially exposed to 4 μg/ml oligomycin to stop ATPsynthase activity, 4 μM FCCP to dissipate mitochondrial membrane potential and potentiate maximum oxidative phosphorylation activity, and 4 μg/ml antimycin A to inhibit complex IV and completely stop mitochondria oxidative phosphorylation. OCR values for each injection step were calculated as the mean of the measurements of two cycles per injection step and used to determine the following parameters: basal respiration (basal mitochondrial respiration before addition of any compound); ATP-linked respiration (basal minus oligomycin-induced rate, respiration rates associated with the production of ATP by the ATP synthase); maximum capacity (FCCP-induced minus antimycin-A-induced rate, maximum respiratory capacity achieved by the activity of complex I–IV); and spare capacity (FCCP-induced minus basal rate, mitochondria capacity above basal levels that can be recruited in situations of high energetic demand).

### Enzymatic assays

Enzymatic assays were performed using the Complex I and IV Enzyme Activity Microplate Assay kits (Abcam) according to the manufacturer's instructions.

### Statistical analysis

Quantitative data was collected using Microsoft Excel. Statistics for pathway analysis and gene ontology enrichments were calculated by their respective software, using the right-tailed Fisher's exact test. All other statistics were performed using GraphPad Prism^®^ (detailed in the results section or figure legends). Statistical significance was considered to be *P*<0.05.

## References

[JCS194241C1] Bergoffen J., Scherer S. S., Wang S., Scott M. O., Bone L. J., Paul D. L., Chen K., Lensch M. W., Chance P. F. and Fischbeck K. H. (1993). Connexin mutations in X-linked Charcot-Marie-Tooth disease. *Science* 262, 2039-2042. 10.1126/science.8266101 8266101

[JCS194241C2] Burre J., Sharma M., Tsetsenis T., Buchman V., Etherton M. R. and Sudhof T. C. (2010). Alpha-synuclein promotes SNARE-complex assembly in vivo and in vitro. *Science* 329, 1663-1667. 10.1126/science.1195227 20798282 PMC3235365

[JCS194241C3] Burré J., Sharma M. and Südhof T. C. (2014). alpha-Synuclein assembles into higher-order multimers upon membrane binding to promote SNARE complex formation. *Proc. Natl. Acad. Sci. USA* 111, E4274-E4283. 10.1073/pnas.1416598111 25246573 PMC4210039

[JCS194241C4] Cabin D. E., Shimazu K., Murphy D., Cole N. B., Gottschalk W., McIlwain K. L., Orrison B., Chen A., Ellis C. E., Paylor R. et al. (2002). Synaptic vesicle depletion correlates with attenuated synaptic responses to prolonged repetitive stimulation in mice lacking alpha-synuclein. *J. Neurosci.* 22, 8797-8807. 12388586 10.1523/JNEUROSCI.22-20-08797.2002 PMC6757677

[JCS194241C5] Chandra S., Gallardo G., Fernández-Chacón R., Schlüter O. M. and Südhof T. C. (2005). Alpha-synuclein cooperates with CSPalpha in preventing neurodegeneration. *Cell* 123, 383-396. 10.1016/j.cell.2005.09.028 16269331

[JCS194241C6] Charbonnier-Beaupel F., Malerbi M., Alcacer C., Tahiri K., Carpentier W., Wang C., During M., Xu D., Worley P. F., Girault J.-A. et al. (2015). Gene expression analyses identify Narp contribution in the development of L-DOPA-induced dyskinesia. *J. Neurosci.* 35, 96-111. 10.1523/JNEUROSCI.5231-13.2015 25568106 PMC6605247

[JCS194241C7] Choi S. W., Gerencser A. A. and Nicholls D. G. (2009). Bioenergetic analysis of isolated cerebrocortical nerve terminals on a microgram scale: spare respiratory capacity and stochastic mitochondrial failure. *J. Neurochem.* 109, 1179-1191. 10.1111/j.1471-4159.2009.06055.x 19519782 PMC2696043

[JCS194241C8] Di Maio R., Barrett P. J., Hoffman E. K., Barrett C. W., Zharikov A., Borah A., Hu X., McCoy J., Chu C. T., Burton E. A. et al. (2016). alpha-Synuclein binds to TOM20 and inhibits mitochondrial protein import in Parkinson's disease. *Sci. Transl. Med.* 8, 342ra78 10.1126/scitranslmed.aaf3634 PMC5016095 27280685

[JCS194241C9] Eaton S. L., Roche S. L., Llavero Hurtado M., Oldknow K. J., Farquharson C., Gillingwater T. H. and Wishart T. M. (2013). Total protein analysis as a reliable loading control for quantitative fluorescent western blotting. *PLoS ONE* 8, e72457 10.1371/journal.pone.0072457 24023619 PMC3758299

[JCS194241C10] Fleming M. D., Campagna D. R., Haslett J. N., Trenor C. C. III and Andrews N. C. (2001). A mutation in a mitochondrial transmembrane protein is responsible for the pleiotropic hematological and skeletal phenotype of flexed-tail (f/f) mice. *Genes Dev.* 15, 652-657. 10.1101/gad.873001 11274051 PMC312659

[JCS194241C11] Fowler S. L., Akins M., Zhou H., Figeys D. and Bennett S. A. L. (2013). The liver connexin32 interactome is a novel plasma membrane-mitochondrial signaling nexus. *J. Proteome Res.* 12, 2597-2610. 10.1021/pr301166p 23590695 PMC3714164

[JCS194241C12] Fuller H. R., Hurtado M. L., Wishart T. M. and Gates M. A. (2014). The rat striatum responds to nigro-striatal degeneration via the increased expression of proteins associated with growth and regeneration of neuronal circuitry. *Proteome Sci.* 12, 20 10.1186/1477-5956-12-20 24834013 PMC4021461

[JCS194241C13] Haelterman N. A., Yoon W. H., Sandoval H., Jaiswal M., Shulman J. M. and Bellen H. J. (2014). A mitocentric view of Parkinson's disease. *Annu. Rev. Neurosci.* 37, 137-159. 10.1146/annurev-neuro-071013-014317 24821430 PMC4659514

[JCS194241C14] Jin J., Davis J., Zhu D., Kashima D. T., Leroueil M., Pan C., Montine K. S. and Zhang J. (2007). Identification of novel proteins affected by rotenone in mitochondria of dopaminergic cells. *BMC Neurosci.* 8, 67 10.1186/1471-2202-8-67 17705834 PMC2000881

[JCS194241C15] Lashuel H. A., Overk C. R., Oueslati A. and Masliah E. (2013). The many faces of alpha-synuclein: from structure and toxicity to therapeutic target. *Nat. Rev. Neurosci.* 14, 38-48. 10.1038/nrn3406 23254192 PMC4295774

[JCS194241C16] Li X., Han D., Kin Ting Kam R., Guo X., Chen M., Yang Y., Zhao H. and Chen Y. (2010). Developmental expression of sideroflexin family genes in Xenopus embryos. *Dev. Dyn.* 239, 2742-2747. 10.1002/dvdy.22401 20737508

[JCS194241C17] Liu G., Zhang C., Yin J., Li X., Cheng F., Li Y., Yang H., Uéda K., Chan P. and Yu S. (2009). alpha-Synuclein is differentially expressed in mitochondria from different rat brain regions and dose-dependently down-regulates complex I activity. *Neurosci. Lett.* 454, 187-192. 10.1016/j.neulet.2009.02.056 19429081

[JCS194241C18] McGorum B. C., Pirie R. S., Eaton S. L., Keen J. A., Cumyn E. M., Arnott D. M., Chen W., Lamont D. J., Graham L. C., Llavero Hurtado M. et al. (2015). Proteomic profiling of cranial (Superior) cervical ganglia reveals beta-amyloid and ubiquitin proteasome system perturbations in an equine multiple system neuropathy. *Mol. Cell. Proteomics* 14, 3072-3086. 10.1074/mcp.M115.054635 26364976 PMC4638047

[JCS194241C19] Minjarez B., Calderón-González K. G., Rustarazo M. L. V., Herrera-Aguirre M. E., Labra-Barrios M. L., Rincon-Limas D. E., Del Pino M. M. S., Mena R. and Luna-Arias J. P. (2016). Identification of proteins that are differentially expressed in brains with Alzheimer's disease using iTRAQ labeling and tandem mass spectrometry. *J. Proteomics* 139, 103-121. 10.1016/j.jprot.2016.03.022 27012543

[JCS194241C20] Murphy D. D., Rueter S. M., Trojanowski J. Q. and Lee V. M. (2000). Synucleins are developmentally expressed, and alpha-synuclein regulates the size of the presynaptic vesicular pool in primary hippocampal neurons. *J. Neurosci.* 20, 3214-3220. 10777786 10.1523/JNEUROSCI.20-09-03214.2000 PMC6773130

[JCS194241C21] Nakamura K. (2013). alpha-Synuclein and mitochondria: partners in crime? *Neurotherapeutics* 10, 391-399. 10.1007/s13311-013-0182-9 23512373 PMC3701775

[JCS194241C22] Nemani V. M., Lu W., Berge V., Nakamura K., Onoa B., Lee M. K., Chaudhry F. A., Nicoll R. A. and Edwards R. H. (2010). Increased expression of alpha-synuclein reduces neurotransmitter release by inhibiting synaptic vesicle reclustering after endocytosis. *Neuron* 65, 66-79. 10.1016/j.neuron.2009.12.023 20152114 PMC3119527

[JCS194241C23] Nishimura N., Gotoh T., Oike Y. and Yano M. (2014). TMEM65 is a mitochondrial inner-membrane protein. *PeerJ* 2, e349 10.7717/peerj.349 24765583 PMC3994636

[JCS194241C24] Pagliarini D. J., Calvo S. E., Chang B., Sheth S. A., Vafai S. B., Ong S.-E., Walford G. A., Sugiana C., Boneh A., Chen W. K. et al. (2008). A mitochondrial protein compendium elucidates complex I disease biology. *Cell* 134, 112-123. 10.1016/j.cell.2008.06.016 18614015 PMC2778844

[JCS194241C25] Parihar M. S., Parihar A., Fujita M., Hashimoto M. and Ghafourifar P. (2008). Mitochondrial association of alpha-synuclein causes oxidative stress. *Cell. Mol. Life Sci.* 65, 1272-1284. 10.1007/s00018-008-7589-1 18322646 PMC11131648

[JCS194241C26] Sanhueza M., Chai A., Smith C., McCray B. A., Simpson T. I., Taylor J. P. and Pennetta G. (2015). Network analyses reveal novel aspects of ALS pathogenesis. *PLoS Genet.* 11, e1005107 10.1371/journal.pgen.1005107 25826266 PMC4380362

[JCS194241C27] Scott D. and Roy S. (2012). alpha-Synuclein inhibits intersynaptic vesicle mobility and maintains recycling-pool homeostasis. *J. Neurosci.* 32, 10129-10135. 10.1523/JNEUROSCI.0535-12.2012 22836248 PMC3426499

[JCS194241C28] Simunovic F., Yi M., Wang Y., Macey L., Brown L. T., Krichevsky A. M., Andersen S. L., Stephens R. M., Benes F. M. and Sonntag K. C. (2009). Gene expression profiling of substantia nigra dopamine neurons: further insights into Parkinson's disease pathology. *Brain* 132, 1795-1809. 10.1093/brain/awn323 19052140 PMC2724914

[JCS194241C29] Specht C. G. and Schoepfer R. (2001). Deletion of the alpha-synuclein locus in a subpopulation of C57BL/6J inbred mice. *BMC Neurosci.* 2, 11 10.1186/1471-2202-2-11 11591219 PMC57740

[JCS194241C30] Stefanis L. (2012). α-synuclein in Parkinson's disease. *Cold Spring Harb. Perspect. Med.* 2, a009399 10.1101/cshperspect.a009399 22355802 PMC3281589

[JCS194241C31] Wishart T. M., Rooney T. M., Lamont D. J., Wright A. K., Morton A. J., Jackson M., Freeman M. R. and Gillingwater T. H. (2012). Combining comparative proteomics and molecular genetics uncovers regulators of synaptic and axonal stability and degeneration in vivo. *PLoS Genet.* 8, e1002936 10.1371/journal.pgen.1002936 22952455 PMC3431337

